# Supplementary material for: Geospatial modeling and forecasting of urban land use change using Google Earth Engine and machine learning
Source: PLoS One. 2025 Dec 18;20(12):e0338920. doi: 10.1371/journal.pone.0338920 (PMC12714270; doi:10.1371/journal.pone.0338920)
Supplement: S1 Table — (PDF) [file pone.0338920.s001.pdf]

LULC Change Matrices and Descriptions

Karachi

Karachi's change matrix highlights intensive barren-to-urban transitions (30.5%), making it the dominant contributor to built-up expansion. Vegetation also suffered significant conversion into urban and barren categories. Water resources decreased sharply, with over 6% converted to urban areas. These transitions explain Karachi's explosive growth of +105% urban land, while exposing severe ecological strain in Pakistan's largest city.

S1 Table. LULC Change Matrix for Karachi (1990–2020) in % of Total Area.

From \ To	Urban	Vegetation	Water	Barren	Total Loss
Urban	—	2.1	0.5	1.6	4.2
Vegetation	28.4	—	3.0	10.7	42.1
Water	6.2	2.4	—	1.5	10.1
Barren	30.5	9.4	3.7	—	43.6
Total Gain	65.1	13.9	7.2	13.8	100
