# Supplementary material for: Geospatial modeling and forecasting of urban land use change using Google Earth Engine and machine learning
Source: PLoS One. 2025 Dec 18;20(12):e0338920. doi: 10.1371/journal.pone.0338920 (PMC12714270; doi:10.1371/journal.pone.0338920)
Supplement: S2 Table — (PDF) [file pone.0338920.s002.pdf]

LULC Change Matrices and Descriptions

Islamabad

Islamabad shows a more moderate urban expansion pattern compared to Karachi and Quetta. Vegetation-to-urban transitions (29.5%) dominate, followed by barren-to-urban conversions. Although water loss is less dramatic than in Lahore, steady conversion into urban areas remains evident. These results underline how even planned capital expansion exerts continuous pressure on ecological systems.

S2 Table. LULC Change Matrix for Islamabad (1990–2020) in % of Total Area.

From \ To	Urban	Vegetation	Water	Barren	Total Loss
Urban	—	1.6	0.3	0.8	2.7
Vegetation	29.5	—	1.7	14.3	45.5
Water	4.8	2.1	—	0.9	7.8
Barren	23.2	11.0	2.1	—	36.3
Total Gain	57.5	14.7	4.1	15.0	100
