# Supplementary material for: Geospatial modeling and forecasting of urban land use change using Google Earth Engine and machine learning
Source: PLoS One. 2025 Dec 18;20(12):e0338920. doi: 10.1371/journal.pone.0338920 (PMC12714270; doi:10.1371/journal.pone.0338920)
Supplement: S3 Table — (PDF) [file pone.0338920.s003.pdf]

LULC Change Matrices and Descriptions

Peshawar

Peshawar exhibits a balanced transition profile, with barren-to-urban and vegetation-to-urban conversions accounting for most urban growth. Interestingly, some vegetation gains occurred in hilly areas, although these were offset by losses elsewhere. Water resources decreased steadily, with nearly 4% lost to urban use. These results show that while Peshawar retained pockets of greenery, water scarcity remains a critical issue.

S3 Table. LULC Change Matrix for Peshawar (1990–2020) in % of Total Area.

From \ To	Urban	Vegetation	Water	Barren	Total Loss
Urban	—	1.9	0.2	0.9	3.0
Vegetation	22.7	—	2.0	11.5	36.2
Water	3.9	2.7	—	1.2	7.8
Barren	25.5	12.1	2.4	—	40.0
Total Gain	52.1	16.7	4.6	15.7	100
