# Supplementary material for: Geospatial modeling and forecasting of urban land use change using Google Earth Engine and machine learning
Source: PLoS One. 2025 Dec 18;20(12):e0338920. doi: 10.1371/journal.pone.0338920 (PMC12714270; doi:10.1371/journal.pone.0338920)
Supplement: S4 Table — (PDF) [file pone.0338920.s004.pdf]

LULC Change Matrices and Descriptions

Quetta

Quetta demonstrates the most dramatic transformation, with over 36% of barren land converted to urban areas and a 404% increase in total urban land. Vegetation and water bodies were also drastically reduced, further aggravating the city's ecological vulnerability. These changes reflect Quetta's constrained high-altitude basin geography, where rapid urbanization has encroached aggressively on natural resources.

S4 Table. LULC Change Matrix for Quetta (1990–2020) in % of Total Area.

From \ To	Urban	Vegetation	Water	Barren	Total Loss
Urban	—	2.2	0.6	1.3	4.1
Vegetation	26.4	—	1.9	11.7	40.0
Water	4.2	3.1	—	1.6	8.9
Barren	36.7	10.8	2.4	—	49.9
Total Gain	67.3	16.1	4.9	11.7	100
