# Supplementary material for: Geospatial modeling and forecasting of urban land use change using Google Earth Engine and machine learning
Source: PLoS One. 2025 Dec 18;20(12):e0338920. doi: 10.1371/journal.pone.0338920 (PMC12714270; doi:10.1371/journal.pone.0338920)
Supplement: S4 Fig — Panels (a–d) show classified maps for 1990, 2000, 2010, and 2020. Panels (e–g) depict decadal changes. Panel (h) presents percentage changes in Water, Urban Land, Vegetation, and Barren Land, highlighting significant urban expansion and natural resource decline. (PDF) [file pone.0338920.s008.pdf]

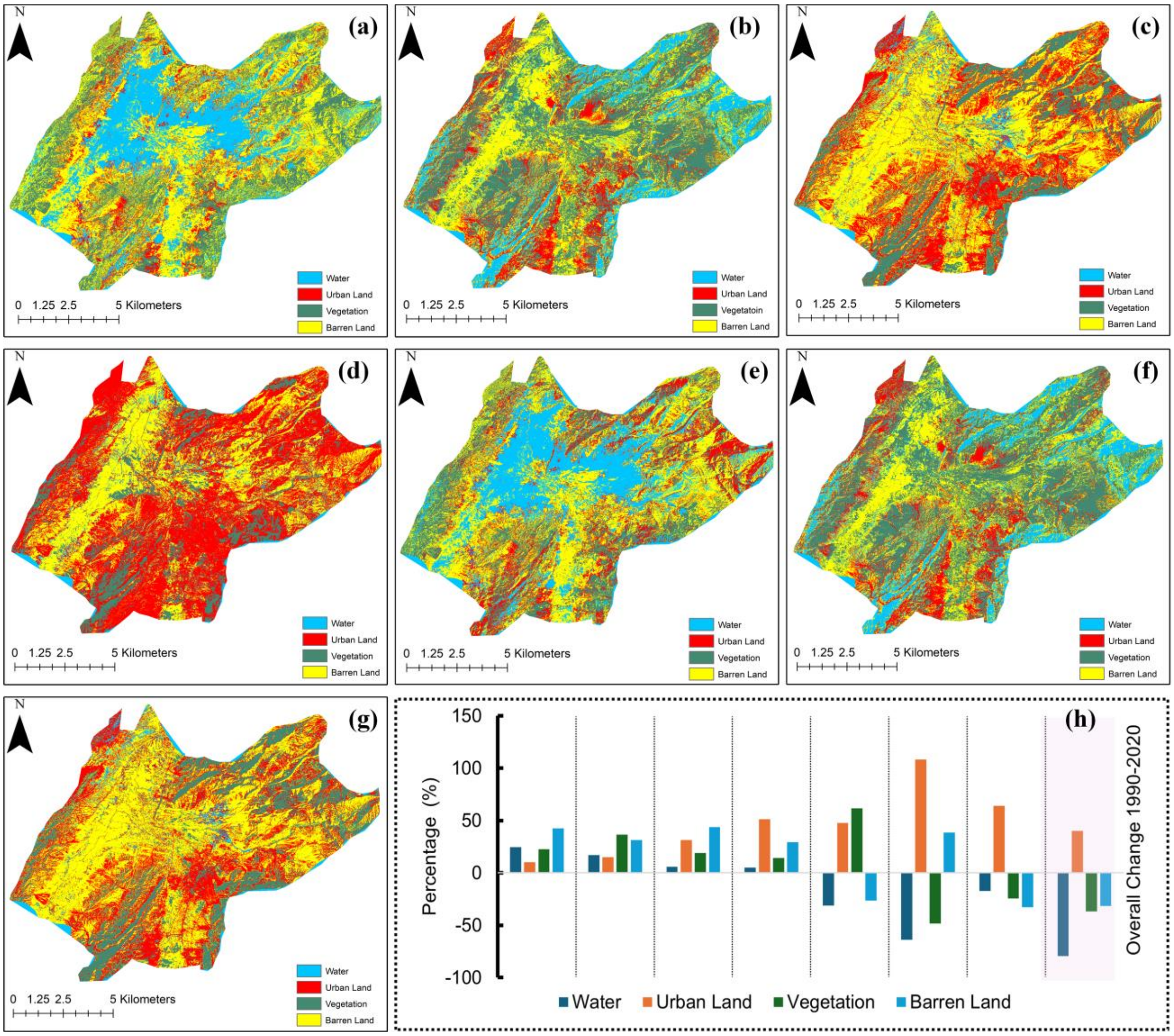

S4 Fig. LULC changes in Quetta from 1990 to 2020. Panels (a–d) show classified maps for 1990, 2000, 2010, and 2020. Panels (e–g) depict decadal changes. Panel (h) presents percentage changes in Water, Urban Land, Vegetation, and Barren Land, highlighting significant urban expansion and natural resource decline.
