# Supplementary material for: Geospatial modeling and forecasting of urban land use change using Google Earth Engine and machine learning
Source: PLoS One. 2025 Dec 18;20(12):e0338920. doi: 10.1371/journal.pone.0338920 (PMC12714270; doi:10.1371/journal.pone.0338920)
Supplement: Attachment — Submitted filename: Response File.docx [file pone.0338920.s010.docx]

**Review Comments to the Author**

Reviewer #1: This paper deals with an important aspect it studies “Geospatial Modeling and Forecasting of Urban Land Use Change Using Google Earth Engine and Machine Learning”. However, the following comments can improve the paper:

(a) The abstract should be quantitative

We thank the reviewer for pointing this out. In the revised manuscript, the abstract has been updated to include quantitative details of our findings, including the classification accuracy (74–92%), urban growth percentages (e.g., Quetta +404%, Karachi +105%), vegetation loss (e.g., Islamabad −71%, Karachi −81%), and water decline (e.g., Lahore −91%). Additionally, forecasted urban expansion trends to 2030 (15–25%) are now reported. We believe this quantitative emphasis strengthens the clarity and impact of the abstract.

(b) LULC maps needs improvement.

We thank the reviewer for this valuable feedback. In the revised manuscript, all LULC maps have been improved for clarity, readability, and consistency. A standardized color scheme has been applied (urban = red, vegetation = green, water = blue, barren = yellow), and all figures now include legends, north arrows, and scale bars. To facilitate comparison, maps have been arranged in panel layouts showing temporal changes for each city side by side. All figures were re-exported at 300 dpi resolution to meet publication standards. Additionally, Figure 1 (Study Area) was revised to clearly show (a) the location of the five study cities Karachi, Quetta, Peshawar, Lahore, and Islamabad within Pakistan’s administrative boundaries, and (b) the national LULC distribution. These improvements ensure that the maps are both visually clear and scientifically informative, directly addressing the reviewer’s concern.

(c) Add importance of Elevation on change of LULC

We thank the reviewer for this valuable suggestion. We have now added a dedicated discussion on the role of elevation in LULC change. Specifically, we emphasize how low-lying cities such as Karachi and Lahore facilitate rapid urban expansion, while high-altitude Quetta (1,680m) exhibits more constrained growth patterns due to surrounding hills. We also discuss the influence of elevation on vegetation persistence in Peshawar and agricultural conversion in Lahore. Relevant literature has been cited to support this addition.

(d) Change Matrix needs to be provided

We thank the reviewer for this important suggestion. In the revised manuscript, we have added detailed LULC change matrices for all five study cities (Karachi, Lahore, Islamabad, Peshawar, and Quetta) in the Results section. These matrices quantify the transitions between the four major land cover classes (urban, vegetation, water, and barren) from 1990 to 2020.

The inclusion of these tables allows us to explicitly highlight the major contributors to urban expansion. For example, in Lahore, more than one-third of vegetation was converted into urban areas, while in Karachi, barren-to-urban transitions accounted for the largest share of expansion. Islamabad displayed more moderate but steady vegetation-to-urban transitions, while Peshawar retained some vegetation pockets but lost water resources to urban use. Quetta exhibited the most dramatic transformation, with over one-third of barren land converted into urban areas, resulting in a 404% increase in built-up land.

These additions significantly strengthen the quantitative interpretation of land cover dynamics. The matrices complement our LULC maps and provide clearer evidence of the mechanisms driving urban expansion in Pakistan’s major cities. Decade-wise transition matrices are also included in the supplementary material to provide further detail.

(e) Why is the preference of choosing RF algorithm

We appreciate the reviewer’s query regarding our choice of the Smile Random Forest (SRF) algorithm. In the revised manuscript, we have clarified this preference. SRF was selected because it offers several advantages over alternative classifiers: it performs well with large, high-dimensional datasets, is less prone to overfitting, and provides both internal error estimation and variable importance measures. Moreover, when applied within Google Earth Engine, SRF achieved the highest overall classification accuracy (74–92%) compared to other classifiers tested (e.g., CART, SVM). These strengths, along with consistent validation in prior remote sensing studies, justified our preference for SRF as the most robust classifier for long-term LULC change analysis in diverse urban environments.

(f) The paper needs to clearly mention the novelty

We thank the reviewer for highlighting this point. In the revised manuscript, we have explicitly added a statement of novelty at the end of the Introduction. The novelty lies in (i) the integration of Smile Random Forest with Google Earth Engine for long-term LULC classification, (ii) the multi-decadal analysis of five major Pakistani cities combined with predictive modeling to 2030, (iii) robust validation using accuracy and kappa metrics, and (iv) direct linkage of findings with Sustainable Development Goals (SDGs). This explicit articulation of novelty clarifies the unique contributions of our study relative to previous work.

(g) The paper needs to be connected to the Food Security

We thank the reviewer for this insightful observation. In the revised manuscript, we have explicitly connected LULC changes with food security. A new paragraph has been added in the Discussion section, explaining how the conversion of agricultural land and vegetation into urban settlements, coupled with water loss, poses risks to food availability and agricultural productivity. Additionally, the Conclusion now emphasizes the importance of integrating LULC monitoring with food security planning. These additions strengthen the societal relevance of the study by linking urban expansion to one of Pakistan’s most pressing challenges.

(h) The result and discussion section needs to compare and other study facts and figures with the present papers statistics

We thank the reviewer for this valuable suggestion. In the revised manuscript, we have strengthened the Results and Discussion section by explicitly comparing our findings with those of prior studies. For example, we show that our results on urban expansion in Lahore and Karachi are consistent with Ahmad et al. (2022) and Baqa et al. (2021), while our projections for Quetta extend beyond previous descriptive studies by quantifying a 404% increase in urban land. We also compare our findings with Patel et al. (2024) in India, showing similar vegetation-to-urban transitions across South Asia. These comparisons highlight both the robustness of our results and the broader regional relevance of our findings.

(i) Prediction of LULC needs to be further checked

We thank the reviewer for this observation. In the revised manuscript, we have strengthened the explanation of our LULC prediction modeling. Specifically, we highlight that the MOLUSCE–ANN approach was validated using cross-validation, Δ Overall Accuracy, and Cohen’s Kappa metrics. The 2030 projections achieved kappa values ranging from 0.80 (substantial agreement) to 0.96 (almost perfect agreement), confirming that the predictive results are statistically robust. We believe this clarification demonstrates that the forecasts presented are not speculative but are grounded in strong statistical validation.

(j) Following papers can be used as references for better understanding and cited:

(i) Geospatial Modeling and Forecasting of Urban Land Use Change Using Google Earth Engine and Machine Learning

(ii) Geospatial Modeling and Forecasting of Urban Land Use Change Using Google Earth Engine and Machine Learning

(iii) Integrating Multi-Source Satellite Imagery and Socio-Economic Household Data for Wealth-Based Poverty Assessment of India: A GIS and Machine Learning Based Approach.

We thank the reviewer for suggesting these important references. We have now cited Geospatial Modeling and Forecasting of Urban Land Use Change Using Google Earth Engine and Machine Learning in both the Introduction and Discussion to contextualize our methodological framework and to compare results. Additionally, we have cited Ji et al. (2022) for multi-source geospatial modeling and Singh et al. (2025) for integrating socio-economic data with satellite imagery in poverty assessment. These insertions strengthen the literature review, position our work in relation to existing studies, and highlight the novelty of our contribution.

Reviewer #2: This manuscript addresses a relevant and timely topic urban land use and land cover (LULC) change in rapidly growing cities in Pakistan using modern remote sensing technologies and cloud-based platforms. Certain terms are used inconsistently or without definition, and several paragraphs suffer from awkward phrasing, overly long sentences, or repetition.

1. The writing is verbose in several places, and multiple sentences contain grammatical or syntactical errors that obscure the meaning.

We thank the reviewer for this observation. We have carefully revised the manuscript to improve clarity, conciseness, and readability. Verbose sentences have been streamlined, and grammatical and syntactical errors have been corrected throughout the paper. These revisions ensure that the meaning is conveyed more clearly and that the overall presentation meets academic writing standards.

2. There is excessive repetition of core concepts such as urban expansion, LULC change, and machine learning algorithms.

3. The novelty claim would be strengthened by clearer differentiation from prior studies, perhaps with a short paragraph detailing how this study exceeds or improves upon earlier efforts.

We thank the reviewer for this constructive suggestion. In the revised manuscript, we have added a dedicated paragraph at the end of the Introduction that explicitly differentiates our work from prior studies in Pakistan and South Asia. We clarify that earlier LULC research has been limited to short-term or single-city analyses and has often relied on conventional classifiers such as CART, SVM, or CA–Markov models. In contrast, our study integrates the Smile Random Forest (SRF) algorithm within the Google Earth Engine framework, covers three decades (1990–2020) across five major cities, and extends the analysis with predictive modeling to 2030 using MOLUSCE–ANN. We also emphasize that the study achieved robust accuracy (74–92% with kappa up to 0.96) and links urban expansion directly to SDGs, thereby providing stronger evidence for sustainable planning. This explicit differentiation clarifies how our work exceeds earlier efforts and highlights the novelty of our contribution.

4. The integration of long-term temporal analysis (1990–2020) with forecasting to 2030 is commendable, but the validation method (e.g., accuracy assessment, transition matrices) is not mentioned in the abstract or introduction.

We thank the reviewer for highlighting this important point. In the revised manuscript, we have explicitly mentioned our validation methods in both the Abstract and the Introduction. The Abstract now states that the SRF classifier achieved accuracies between 74–92% with kappa values up to 0.96, and that predictions were validated through transition matrices and cross-validation. Similarly, the Introduction now notes that the study validates its forecasts using accuracy assessment and change matrices. These additions clarify our methodological rigor and assure readers that the long-term predictions are statistically robust.

5. The objectives are clearly laid out, but the methodological summary is vague in parts. For example, mention of “MOLUSCE” in the introduction is abrupt and unexplained.

We thank the reviewer for this valuable feedback. In the revised manuscript, we have clarified the methodological summary in the Introduction. Specifically, the reference to MOLUSCE has been expanded to explain that it is a QGIS plugin (Modules for Land Use Change Evaluation) that uses cellular automata and transition probability matrices for simulating future land cover scenarios. We have also updated the objectives statement to indicate that MOLUSCE was employed for forecasting LULC changes. These changes ensure that the methodology is introduced more clearly and without abrupt references.

6. The manuscript references a broad range of sources, but the integration of this literature into the narrative is uneven. Some citations appear to be added for completeness rather than supporting a specific argument.

We thank the reviewer for this insightful observation. In the revised manuscript, we have restructured the literature integration to ensure that each reference supports a specific point. For example, the sentence in the Discussion that previously listed algorithms has been revised to clarify the distinct roles of each method: “Support Vector Machines (SVM) and Random Forests (RF) have been widely recognized for their ability to handle high-dimensional remote sensing data with strong classification accuracy, while Classification and Regression Trees (CART) and CA–Markov models have been commonly used to simulate and forecast urban expansion trends [43, 46, 47].” Similar refinements were made throughout the Introduction and Discussion to reduce redundancy, avoid citations added only for completeness, and strengthen the connection between prior studies and the novelty of our work.

7. pixelbased should be “pixel-based”.

We thank the reviewer for noting this. The term has been corrected throughout the manuscript, and all instances of pixelbased have been replaced with the correct form pixel-based. We have also double-checked the manuscript for similar typographical inconsistencies.

8. “multi-temporal Landsat data with a Smile Random Forest classifier” needs clarity.

We thank the reviewer for pointing out this ambiguity. In the revised manuscript, particularly in the Abstract, we have clarified this phrase to explicitly distinguish the dataset from the method. It now reads: “multi-temporal Landsat imagery (1990–2020), which was classified using the Smile Random Forest (SRF) algorithm within the Google Earth Engine platform.” This clarification ensures that the data source and the classification approach are clearly and accurately presented.

9. Define acronyms on first use (e.g., GEE, LULC, SRF).

We thank the reviewer for this helpful reminder. In the revised manuscript, all acronyms have been defined at their first occurrence. For example, Google Earth Engine (GEE), Land Use and Land Cover (LULC), and Smile Random Forest (SRF) are now introduced in full before being abbreviated. We also reviewed the manuscript thoroughly to ensure that all other acronyms follow this standard convention.

10. Terms like “MLAs” and “cyber geospatial infrastructure” are used without explanation and feel disconnected.

We appreciate the reviewer’s feedback. The sentence referring to “cyber geospatial infrastructure” has been revised for clarity. It now reads: “It is necessary to develop tools that support the practical use of cloud-based geospatial infrastructure, enabling more effective management, analysis, visualization, and sharing of large-scale geospatial datasets through high-speed networks.” This revision provides a clearer explanation of the concept and avoids unnecessary jargon.

11. Population statistics are outdated or confusing. You refer to a 2016 census for Islamabad, but Pakistan's last full census was in 2017.

We thank the reviewer for pointing this out. In the revised manuscript, all population statistics have been updated to reflect the official 2017 Pakistan Census conducted by the Pakistan Bureau of Statistics. References to the 2016 figures for Islamabad have been corrected. Where relevant, we also note UN World Urbanization Prospects (2022) estimates to highlight ongoing population growth beyond the last census. These revisions ensure consistency and prevent confusion regarding demographic baselines.

12. Why were only these five cities selected, and why not include emerging urban centers like Faisalabad or Multan?

We thank the reviewer for this important question. In the revised manuscript, we have clarified the rationale for selecting the five study cities. Karachi, Lahore, Islamabad, Peshawar, and Quetta were chosen because they are provincial or federal capitals, each representing unique urbanization patterns, topographies, and socio-economic settings. This selection ensures coverage of Pakistan’s most influential urban centers while capturing diverse land use dynamics across different ecological and demographic contexts. Although other emerging cities such as Faisalabad and Multan are also significant, they were not included in order to focus the study on capital cities for national representativeness. We note in the Discussion that future work should extend this framework to such second-tier cities.

13. Were the city boundaries clearly defined for the study (e.g., administrative boundaries, metropolitan region, or functional urban areas)?

We thank the reviewer for this important observation. In the revised manuscript, we have clarified that the study areas were delineated using official administrative boundary shapefiles obtained from the Pakistan Bureau of Statistics and relevant provincial development authorities. For Karachi and Lahore, metropolitan region shapefiles were used to include peri-urban areas, thereby capturing functional urban growth. This ensures consistency across the five cities while also reflecting realistic extents of urban expansion. The clarification has been added in the Study Area section.

14. No information on model validation, transition rules, or parameter tuning.

We thank the reviewer for this important observation. In the revised manuscript, details on model validation, transition rules, and parameter tuning have been added to Section 3 (Methodology → Prediction and Modeling). These clarifications improve transparency and replicability of our modeling framework.

15. No confusion matrix, Kappa coefficient, F1-score, or user/producer accuracy metrics are provided.

We thank the reviewer for this valuable observation. In the revised manuscript, we have expanded the accuracy assessment to include Cohen’s Kappa coefficient, F1-scores, user’s accuracy, and producer’s accuracy in addition to overall accuracy. A summary example table for Lahore (2020) has been added in the Results section, while a consolidated table is provided in the Supplementary Material, presenting the metrics for all five cities. The results show overall accuracies between 86–90% and Kappa values between 0.86–0.92, with the highest F1-scores for urban and water classes and slightly lower values for vegetation and barren land. These additions provide class-level details and ensure that the classification reliability of the Smile Random Forest approach is reported in a transparent and comprehensive manner.

16. Please ensure all references are from credible journals or institutions. You can consider these references for improvement (https://doi.org/10.1007/s10668-025-06249-6, https://doi.org/10.1038/s41598-024-68492-7, https://doi.org/10.1080/14498596.2024.2364283, https://doi.org/10.1038/s41598-025 87234-x, https://doi.org/10.1007/s10708-024-11217-0, https://doi.org/10.1038/s41598-025-89167-x; https://doi.org/10.1038/s41598-025-87796-w, https://doi.org/10.1038/s41598-025-92034-4).

We thank the reviewer for this valuable suggestion. We have carefully reviewed the reference list to ensure that all citations are drawn from credible, peer-reviewed journals and recognized institutions. The recommended papers provided by the reviewer have also been incorporated into the revised manuscript at appropriate places where they support our arguments. This has strengthened the scientific foundation of the study and aligned the literature base with current, high-quality research.
