# Supplementary material for: Geospatial modeling and forecasting of urban land use change using Google Earth Engine and machine learning
Source: PLoS One. 2025 Dec 18;20(12):e0338920. doi: 10.1371/journal.pone.0338920 (PMC12714270; doi:10.1371/journal.pone.0338920)
Supplement: Attachment — Submitted filename: Response_File_auresp_2.docx [file pone.0338920.s011.docx]

**Review Comments to the Author**

Reviewer #1: This paper deals with an important aspect it studies “Geospatial Modeling and Forecasting of Urban Land Use Change Using Google Earth Engine and Machine Learning”. However, the following comments can improve the paper:

(a) It is mentioned that MOLUCE tool is used then Why QGIS is not mentioned in Methodology diagram

Thank you for your valuable comment. We appreciate your attention to detail. The methodology diagram has now been revised to explicitly include both QGIS and the MOLUSCE plugin as integral components of the LULC prediction workflow. Although QGIS was already described within the methodology section, we acknowledge that it was not reflected in the visual workflow. This update ensures consistency between the textual explanation and the graphical representation.

(b) Which classification system was used for the LULC classification

Thank you for your insightful comment. As clarified in the Methods section, this study employed a four-class hierarchical LULC classification scheme comprising: (a) Urban/Built-up, (b) Vegetation, (c) Water Bodies, and (d) Barren Land. These classes were applied directly in the Smile Random Forest (SRF) classifier within Google Earth Engine. Further details, including class descriptions, are provided in Table 1 of the manuscript. This classification structure aligns with widely adopted practices for Landsat-based urban landscape studies.

(c) How many points were taken to validate the work

Thanks for your comment. We have added the exact counts of training and validation samples (See Methodology Section) in the revised manuscript. In Landsat 8 is a vital satellite for Land Use and Land Cover (LULC) mapping due to its 11 spectral bands and high spatial resolution. Bands 1–7 and Band 9 are multi-spectral with 30 m resolution, Band 8 is panchromatic with 15 m resolution, and Bands 10–11 are thermal infrared with 100 m resolution. Key bands for LULC include Bands 4 (Red), 5 (NIR), and 6 (SWIR1), which are used to differentiate vegetation, urban areas, and soil moisture. Standard indices derived from these bands include NDVI for vegetation health, NDWI for water body detection, and NDBI for identifying built-up areas. These spectral tools enable accurate classification and monitoring of land cover types, including forests, agriculture, urban sprawl, and water bodies, making Landsat 8 indispensable for environmental and urban planning applications.

(d) Compare the results on Map with different algorithms and justify why it was chosen

Thank you for your comment. We have now included a comparative accuracy summary of multiple classification algorithms—CART, SVM, ANN, and SRF in the revised manuscript. The comparison shows that SRF achieved the highest overall accuracy and Kappa coefficient, demonstrating superior class separability and stability across all LULC categories. While the other algorithms performed reasonably well, SRF consistently produced cleaner class boundaries and reduced misclassification, particularly between vegetation and barren land. Based on these performance metrics, SRF was selected as the optimal algorithm for generating the final LULC maps.

(e) The paper needs to clearly mention the novelty

Thank you for your comment. We have added a clearly articulated novelty paragraph in the Introduction. The novelty of this study lies in its integrated, multi-scale, and multi-decadal assessment of urban land-use change across Pakistan using a combined cloud-based and desktop machine-learning workflow. Unlike earlier LULC studies in Pakistan that typically focus on single cities, limited temporal periods, or conventional classifiers (e.g., CART, SVM, CA–Markov), this study:

(i) applies the SRF algorithm within Google Earth Engine to generate harmonized, high-accuracy LULC maps for five major cities over a 30-year period (1990–2020);

(ii) incorporates MOLUSCE–ANN transition modeling in QGIS to predict future urban expansion up to 2030;

(iii) integrates spatial metrics, elevation constraints, and cross-city comparisons to provide a nationally consistent interpretation of urban growth dynamics; and

(iv) explicitly connects LULC transitions with implications for natural resources and food security, offering new SDG-aligned evidence to support sustainable urban planning.

Collectively, this combined analytical framework establishes the first comprehensive, long-term, multi-city urbanization assessment and prediction model for Pakistan.

(f) The paper needs to be connected to the sustainable development goals

Thank you for your comment. We have added a dedicated subsection in the Discussion section explicitly connecting the study’s findings to relevant Sustainable Development Goals (SDGs). The results show that the continued loss of vegetation and water bodies directly impacts SDG 13 (Climate Action) by reducing carbon sequestration potential and increasing exposure to heat stress, flooding, and other climate-related hazards. Likewise, the degradation of natural landscapes and ecosystems aligns with concerns under SDG 15 (Life on Land), as biodiversity and ecological functions become increasingly threatened due to rapid urban expansion.

Additionally, the conversion of productive agricultural land into urban built-up areas particularly pronounced in Lahore, Karachi, and Quetta raises serious implications for SDG 2 (Zero Hunger). The reduction of fertile land and water resources may undermine food production capacity and long-term food security.

Overall, the study demonstrates that prevailing urban growth trajectories pose significant risks to environmental sustainability, climate resilience, ecosystem integrity, and food availability. These insights highlight the urgent need for SDG-aligned urban planning, land management, and policy interventions across Pakistan’s major cities.

(g) The result and discussion section needs to compare and other study facts and figures with the present papers statistics

Thank you for your comment. We have strengthened the Results and Discussion section by incorporating quantitative comparisons with findings from both regional and international studies. These additions highlight how the magnitude and rates of LULC transitions observed in our study align with or differ from previously reported patterns, thereby situating our results within the broader body of urbanization and land-change research. This comparative context enhances the scientific robustness of the discussion and provides clearer justification for the study’s conclusions.

(h) Present a graph present importance of variables used in the study

Thank you for your comment. We have now included a Random Forest Variable Importance plot in the revised manuscript. This figure displays the relative contribution of each predictor variable Landsat spectral bands and key indices such as NDVI, NDBI, SWIR, Red, Green, Blue, and TIRS based on their mean decrease in Gini impurity. The plot provides a clear ranking of variable influence and helps illustrate which spectral features played the most significant role in the LULC classification process.

(i) Following papers can be used as references for better understanding and cited:

(i) Modeling spatio-temporal land use dynamics in Amritsar district, Punjab, India using machine learning

(ii) Unveiling predictive factors for household-level stunting in India: A machine learning approach using NFHS-5 and satellite-driven data

(iii) Ecotope-based diversity monitoring of wetland using infused machine learning technique

(iv) Erratic dynamics of LULC over the temporal window 1978–2017: a case study from western flank of Gulf of Cambay, Gujarat, India

Thank you for the valuable suggestion. We have now incorporated all the recommended references into the revised manuscript. These studies have been cited in the Related Work and Discussion sections to enhance the methodological context and strengthen the scientific foundation of our analysis. Specifically, the following papers were added:

See reference 45: Modeling spatio-temporal land use dynamics in Amritsar district, Punjab, India using machine learning;

See reference 46: Unveiling predictive factors for household-level stunting in India: A machine learning approach using NFHS-5 and satellite-driven data;

See reference 25: Ecotope-based diversity monitoring of wetland using infused machine learning technique; and

See reference 43: Erratic dynamics of LULC over the temporal window 1978–2017: a case study from western flank of Gulf of Cambay, Gujarat, India.

These references provide broader regional insights, methodological parallels, and comparative evidence that collectively improve the depth and relevance of our study.
